# Draft Genome Sequence of *Halomonas elongata* Strain K4, an Endophytic Growth-Promoting Bacterium Enhancing Salinity Tolerance *In Planta*

**DOI:** 10.1128/genomeA.01214-16

**Published:** 2016-11-03

**Authors:** Feras F. Lafi, Juan S. Ramirez-Prado, Intikhab Alam, Vladimir B. Bajic, Heribert Hirt, Maged M. Saad

**Affiliations:** a King Abdullah University of Science and Technology (KAUST), Biological and Environmental Sciences and Engineering Division (BESE), Thuwal, Saudi Arabia; b Computational Bioscience Research Center (CBRC), King Abdullah University of Science and Technology (KAUST), Thuwal, Saudi Arabia

## Abstract

*Halomonas elongata* strain K4 is an endophytic bacterial strain that was isolated from roots of *Cyperus conglomeratus* collected at the Red Sea coast in Thuwal, Saudi Arabia. Here, we present a draft genome sequence of this strain, highlighting a number of pathways involved in plant growth promotion under salt stress.

## GENOME ANNOUNCEMENT

Within the framework of the Darwin21 project (http://www.darwin21.net), an extensive microbial collection of isolates from roots of different desert plants was obtained and revealed a number of strains belonging to the *Halomonadaceae* family that have the ability to promote the growth of *Arabidopsis thaliana* plants under salt stress conditions. Members of the *Halomonadaceae* family are Gram-negative, rod-shaped, and slightly or moderately halotolerant bacteria (1). They are characterized by their metabolic capacity for salt tolerance, including the synthesis of ectoine (2). Ectoine (1,4,5,6-tetrahydro-2-methyl-4-pyrimidine carboxylic acid) is a widely distributed compatible solute accumulating in halophilic and halotolerant microorganisms to prevent osmotic stress in highly saline environments and is responsible for enhancing salt tolerance in bacteria (3, 4) and plants (5–7).

*Halomonas elongata* strain K4 was isolated from surface-sterilized roots of *Cyperus conglomeratus*, a naturally occurring plant in the desert of the Arabian Peninsula. The plants were collected approximately 10 m from the coast of the Red Sea near Thuwal, Saudi Arabia (22.3095°N, 39.1047°E). The root extracts were plated on R2A media (8) supplemented with 3% NaCl. Single colonies were subcultured after selection from a 10^−4^ dilution plate grown at 28°C. Based on 16S rRNA gene analysis, the K4 strain was closely related to both *H. elongata* 1H9 (NR_074782) and *H. elongata* DSM 2581 (NC_014532) with 99% sequence similarity (2). Genomic DNA of the K4 strain was extracted using Qiagen’s DNeasy blood and tissue kit following the manufacturer’s protocol. The DNA library was constructed as described previously (9) and sequenced by paired-end Illumina MiSeq. Contig assembly was done with Spades assembler version 3.6 with a 1-kb contig cutoff size (10). *De novo* assembly of the MiSeq reads for *H. elongata* strain K4 resulted in 35 contigs with a total length of 3,469,874 bp and a mean contig size of ~99,139 bp. The *N*_50_ was 272,670 bp and the *L*_50_ was reached by 4 contigs, with an average GC content of 63.4%. MegaBLAST searches (11) of the K4 concatenated genome against the NCBI reference genome database (http://www.ncbi.nlm.nih.gov/genome) revealed that the closest relative genome was *H. elongata* DSM 2581 (NC_014532) with 87% sequence coverage and 95% sequence identity.

Genome annotation was carried out with the Indigo pipeline (12) with the exception of open reading frame (ORF) prediction by FragGeneScan (13). The annotation of *H. elongata* strain K4 resulted in 2,354 ORFs, 5 rRNAs, 59 tRNAs, and 39 ncRNAs. Analysis of the genome indicated the presence of a single copy of the *ectABC* operon (14) involved in the biosynthesis of ectoine. The genome coded for the three main enzymes involved in the ectoine pathway, namely, L-2,4-diaminobutyric acid acetyltransferase (EctA) (EC: 2.3.1.178), L-2,4-diaminobutyric acid aminotransferase (EctB) (EC: 2.6.1.76), and L-ectoine synthase (EctC) (EC: 4.2.1.108). An additional gene *ectD* coding for ectoine hydroxylase (EC: 1/14/11) is present, and this enzyme converts ectoine to hydroxyectoine (15). Furthermore, the genome encoded other osmoprotectant-related enzymes such as trehalose phosphatase (EC:3.1.3.12) (16) and mannitol-1-phosphate 5-dehydrogenase (EC:1.1.1.17) (17), suggesting a possible role for this endophyte in enhancing salinity tolerance in plants.

### Accession number(s).

The genome sequence of *H. elongata* strain K4 was deposited at DDBJ/EMBL/GenBank under the accession number LWGO00000000. The version described in this paper is the first version, LWGO01000000.
